# Supplementary material for: Prophylactic Multi-Subunit Vaccine against Chlamydia trachomatis: In Vivo Evaluation in Mice
Source: Vaccines (Basel). 2021 Jun 6;9(6):609. doi: 10.3390/vaccines9060609 (PMC8226540; doi:10.3390/vaccines9060609)
Supplement: Supplementary file 1 [file vaccines-09-00609-s001.zip › vaccines-1227884-supplementary.pdf]

vaccines

MDPI

Supplementary Material

Table S1. Clinical scoring (mouse pneumonia).

Parameter	Clinical Observation	Points
Piloerection	Normal coated fur	0
	Partial ruffed fur	1
	Slightly ruffed fur	2
	Ruffed fur	3
Body posture	Normal posture	0
	Slightly hunched posture	1
	Hunched posture	2
Locomotion	Spontaneous movement, movement after cage opening or provocation of sleeping animals, normal behavior	0
	Movement only after provocation	1
	No movement – **humane endpoint**	2
Agility	Normal and fast movements	0
	Slow and / or sluggish	1
Breathing (Pneumoniae only)	Unaffected	0
	Tachypnea	1
	Tachypnea with abdominal effort while breathing, gasping	2
Reaction to tactile stimulation	Normal attentiveness, escape reflex at approach	0
	Attentiveness slightly affected, reduced reaction to external stimuli	1
	Somnolent – **humane endpoint**	2
Ocular or nostril discharge	None	0
	Presence of ocular and / or nostril discharge (→ treatment with eye ointment)	1
	Conjunctivitis with thick ocular discharge (→ treatment with eye ointment)	2
Dehydration (exsiccosis)	Regular skin turgor	0
	Reduced skin turgor, delayed spread of skin fold (→ mashed and soaked wet food on cage floor)	1
	Poor skin turgor, no spread of skin fold – **humane endpoint**	2
Body weight loss	0-3%	0
	3-10%	1
	10-20%	2
	>20%	3
Sum (Pneumoniae)	Sum of clinical score ≥ 14 – **humane endpoint**	Σ19
	**Control frequency: daily**	

Table S2. Mouse group sizes per experiment and C.tr. serovar of Figures 2-8. N for body weight (BW) and clinical score (CS): indicating the number of animals at the beginning of the respective experiment. With few exceptions, all animals surviving until day 7 p.i. were analyzed for their bacterial load (IFU). N for MPO, TNF-α and IFN-γ: For practical reasons, not all surviving animals from larger groups were always analysed. In this case, samples were chosen randomly, *i.e.* without consideration of other parameters/results.

	**Serovar E**			**Serovar D**			**Serovar L2**			**Serovar A**		**Mock**
**Fig.**	**5cVAC**	**Adj.**	**Buffer**	**5cVAC**	**Adj.**	**Buffer**	5cVAC	Adj.	Buffer	5cVAC	Buffer	
**2**	9	8	8	11	10	10	9	10	7	7	7	8
**3** (IFU)	9	8	6	9	9	8	9	10	6	7	4	8
**3** (MPO)	9	8	6	7	7	6	9	-	6	7	4	8
**4**	9	8	6	7	7	7	7	-	6	7	4	8
**5** (BW + CS)	11	11	11									
**5** (IFU)	9	10	10									8
**5** (MPO)	5	5	5									8
**5** (TNF/ IFN)	7	-	5									8

	**Serovar E**				**Mock**
**Fig.**	5cVAC	Adj.	Buffer	2cVAC	
**8** (BW + CS)	9	8	9	9	
**8** (IFU)	9	6	7	7	4
**8** (MPO)	9	7	9	9	8
**8** (TNF/ IFN)	9	7	9	9	8

Table S3. Mouse group sizes per experiment and C.tr. serovar of Figures 9-13. N for body weight (BW) and clinical score (CS): indicating the number of animals at the beginning of the respective experiment. With few exceptions, all animals surviving until day 7 p.i. were analyzed for their bacterial load (IFU). N for MPO, TNF-α and IFN-γ: For practical reasons, not all surviving animals from larger groups were always analysed. In this case, samples were chosen randomly, *i.e.* without consideration of other parameters/results.

	**IgA / M / G**			
**Fig.**	i.n. 5cVAC Donors	s.c. 5cVAC Donors	Adj. Donors	Untreated Donors
**9**	13	10	12	14
	i.n. 5cVAC Recip.	s.c. 5cVAC Recip.	Adj. Recipient	Untreated Recipient
**9**	7	5	6	6

	**Serovar E**				**Mock**
**Fig.**	i.n. 5cVAC Recip.	s.c. 5cVAC Recip.	Adj. Recip.	Untreated Recipient	
**10** (BW + CS)	7	5	6	6	
**10** (IFU)	6	3	6	4	4
**10** (MPO)	6	-	6	-	8
**10** (TNF/ IFN)	6	3	6	4	4

	**Serovar E**			**Mock**
**Fig.**	5cVAC	Adj.	Buffer	
**11** (BW + CS)	9	7	9	
**11** (IFU)	9	7	9	5
**11** (MPO)	9	7	9	4
**11** (TNF/ IFN)	9	7	9	4

	**No C.tr. E challenge**			**7d after C.tr. E challenge**		
**Fig.**	IgA / M / G	IgG1 / 2a / 2b	Non-vac.	IgA / M / G	IgG1 / 2a / 2b	Non-vac.
**12**	5	5	6	6	6	7

	**No C.tr. E challenge**			**7d after C.tr. E challenge**		
**Fig.**	5cVAC	Adjuvant	Buffer	5cVAC	Adjuvant	Buffer

**13***	5	2	4	7	5	5	* splenocyte data obtained from n mice

a

protein	full-length protein [aa]	protein fragment used for vaccination [aa position]	fragment length [aa]	calculated molecular weight [kDa]
PmpA	975	53 - 665	612	67,8
PmpD	1530	34 - 619	585	61,2
PmpG	1013	29 - 673	644	68,5
PmpH	1016	26 - 690	664	70
Ctad1	433	-	433	49

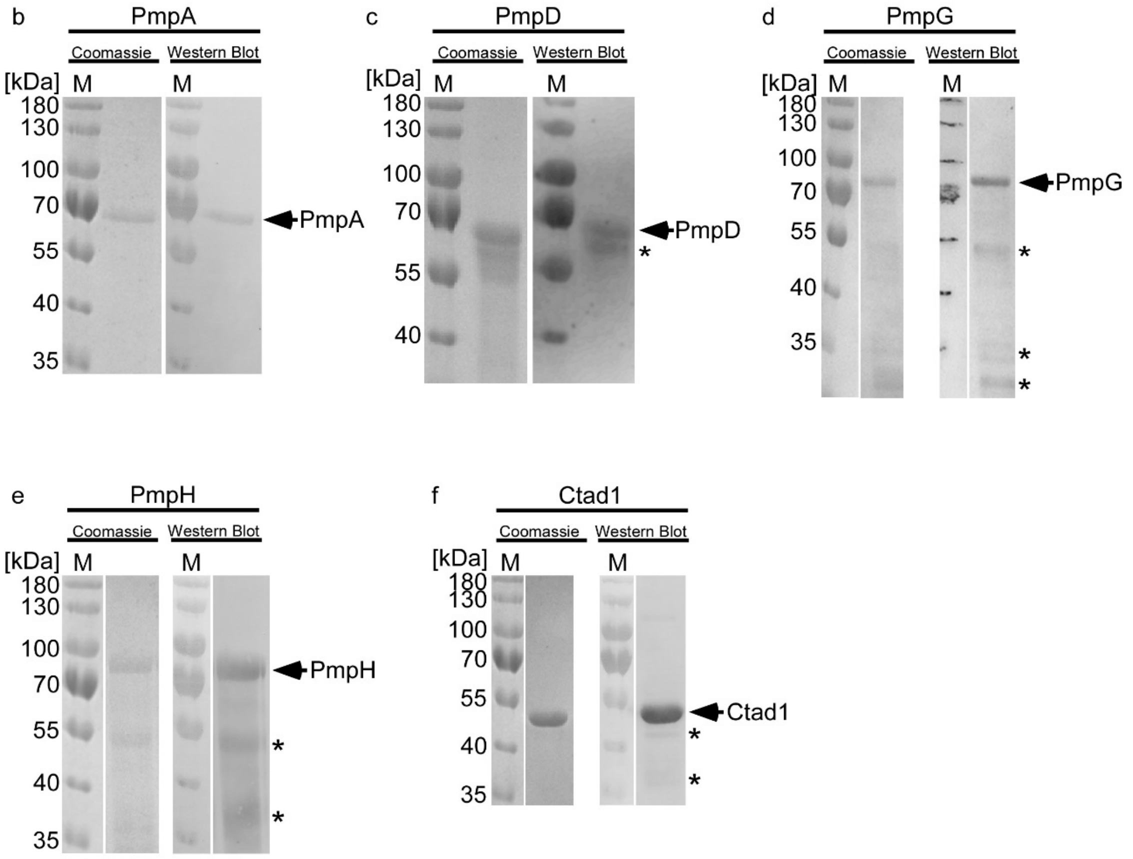

Figure S1. Recombinant proteins used in the 2cVAC and 5cVAC vaccine formulation. (**a**) Table specifying details of recombinant proteins and protein fragments used in this study. The calculated molecular weight numbers given include the HIS-tag. [aa] = Amino acid. (**b**-**f**) Visualization of recombinant proteins, eluted from NiNT agarose column, typically used in the vaccination trial, by Coomassie-stained SDS-Page and Western blot. Protein samples were mixed with protein blue marker and DTT, boiled, and loaded onto 10% SDS gels and subsequently separated. For Coomassie staining, the gel was washed and then heated in a Coomassie solution and incubated at room temperature for the appropriate time. For visualization by Western blot, the proteins were transferred from the gel to a polyvinylidene fluoride (PVDF) membrane. The proteins were subsequently detected with an anti-histidine antibody (1:2,500, Qiagen) and AP-coupled anti-mouse secondary antibodies (1:30,000, Sigma). PageRuler was used as protein marker (M). Protein sizes are given in [kDa]. Stars (*) indicate protein degradation.

Supplement to supplement S1. Uncropped Western Blots

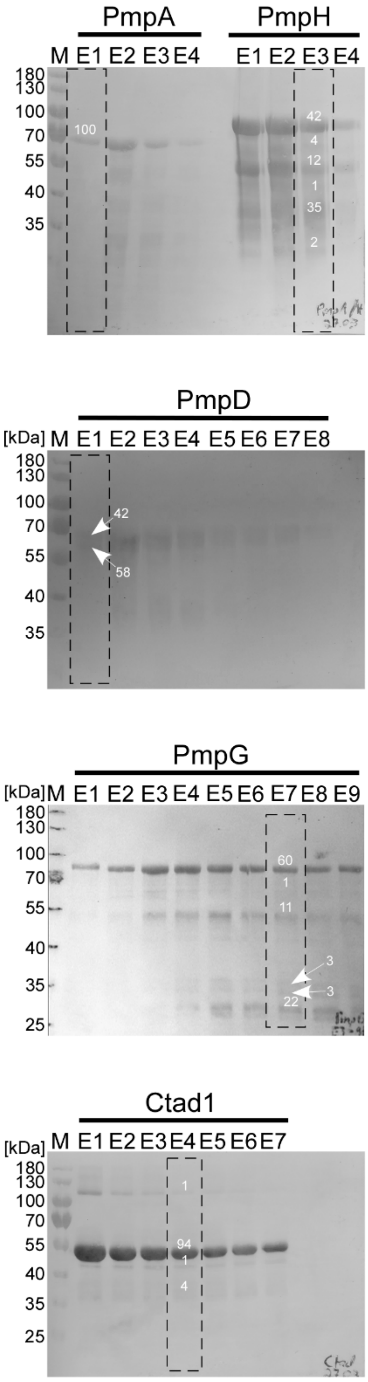

Scheme 1. Uncropped Western blots to those shown in cropped form in Suppl. Figure. S1. Western blots show purified recombinant PmpA, PmpH, PmpD, PmpG and Ctad1 proteins obtained by Ni-NTA affinity chromatography (elution fractions E1 to E9). Elution fractions marked by dashed lines refer to the corresponding lanes in Fig. S1. The Western blots were used to determine the percentage of full-length recombinant protein and degradation products. The intensity of each band detected by the anti-His tag antibody was determined using the software GelAnalyzer2010a and given as percentage of total protein (white numbers [%]). Protein marker (M): PageRuler in kDa.

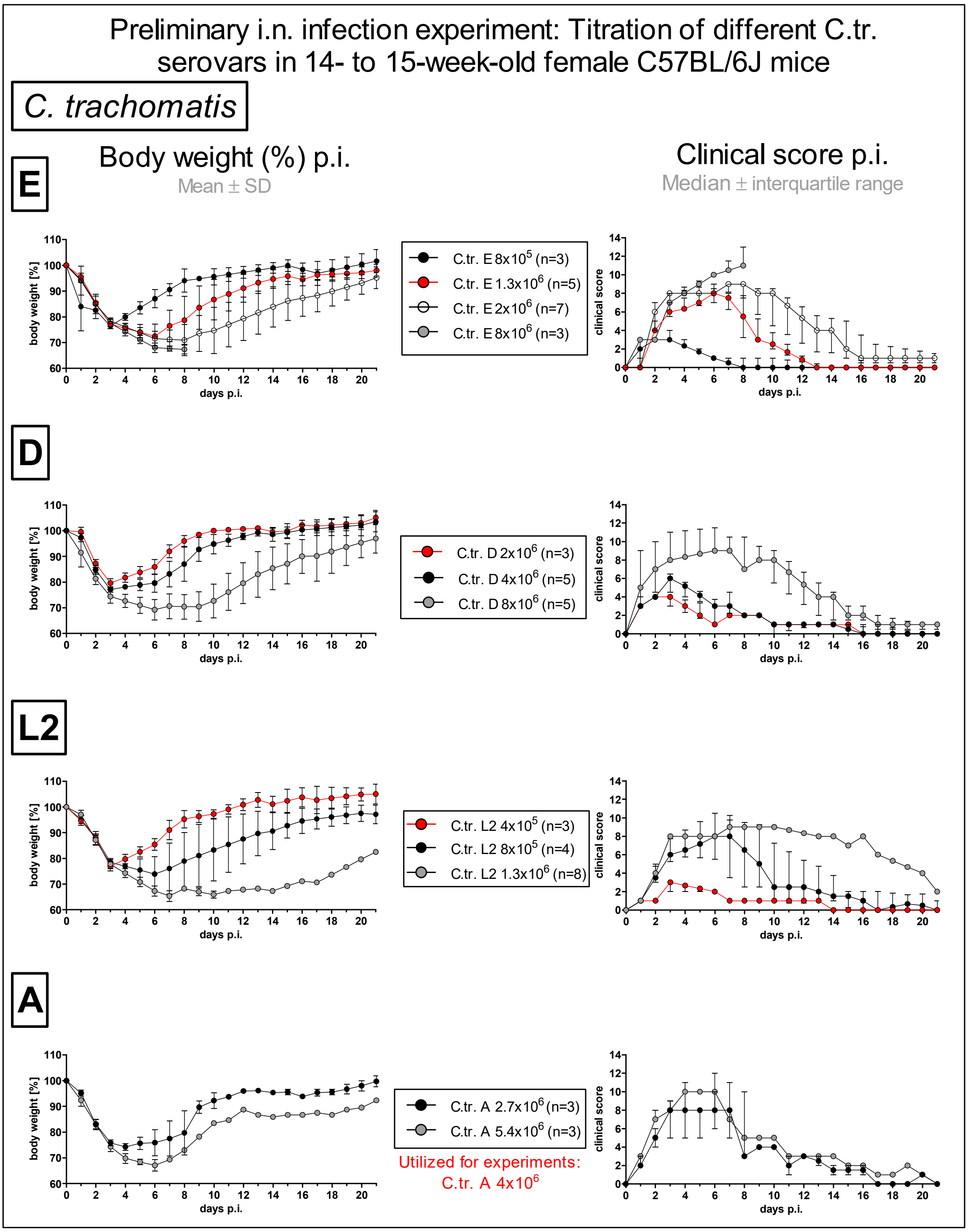

Figure S2. Body weight and clinical score p.i. of an orientating i.n. C.tr. titration experiment for identification of the optimal amount of each serovar using 14- to 15-week-old female, hormone-synchronized mice. In preparation for the mouse lung infection used in the short-term protection model (see Material & Methods and Figure 1a), 14- to 15-week old female hormone-synchronized C57BL/6J mice were infected i.n. with different IFU amounts of C.tr. serovars E, D, L2, and A (as indicated in the boxes). During the following 21 days, body weight [%] (left panels, mean ± standard deviation) and clinical score (right panels, median ± interquartile range) were assessed daily. Red data points and lines depict the C.tr. IFU later used in vaccination experiments. Clinical score and humane endpoint criteria were determined according to Suppl. Material Table S1.

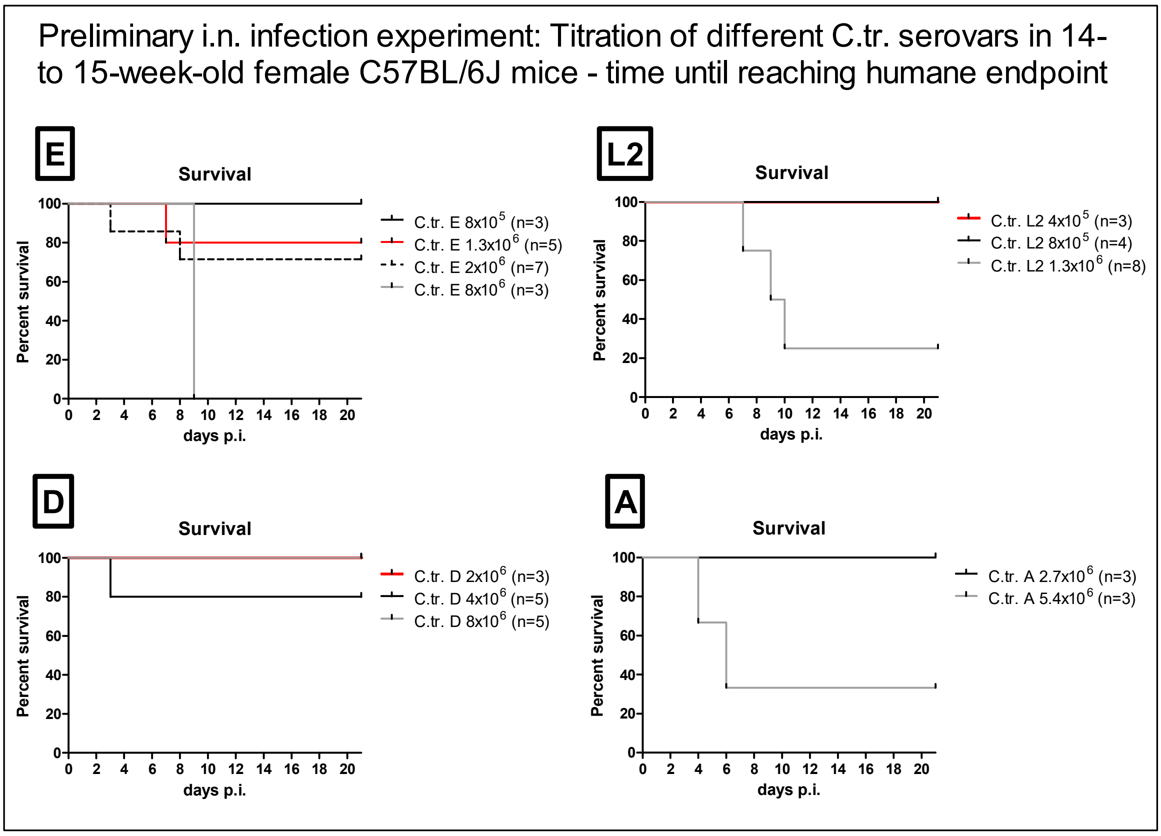

Figure S3. Kaplan-Meier-Plot (survival) of an orientating i.n. C.tr. titration experiment for identification of the optimal amount of each serovar using 14- to 15-week-old female, hormone-synchronized mice. In preparation for the mouse lung infection used in the short-term protection model (see Material & Methods and Figure 1a), 14- to 15-week old female hormone-synchronized C57BL/6J mice were infected i.n. with different IFU amounts of C.tr. serovars E, D, L2, and A (as indicated next to the graphs). During the following 21 days, mice were monitored daily and euthanized painlessly should humane endpoint criteria be reached (see Suppl. Material Table S1). Survival rates [%] of the different serovar and IFU groups are depicted in a Kaplan-Meier-Plot. Red lines depict the C.tr. IFU later used in vaccination experiments.

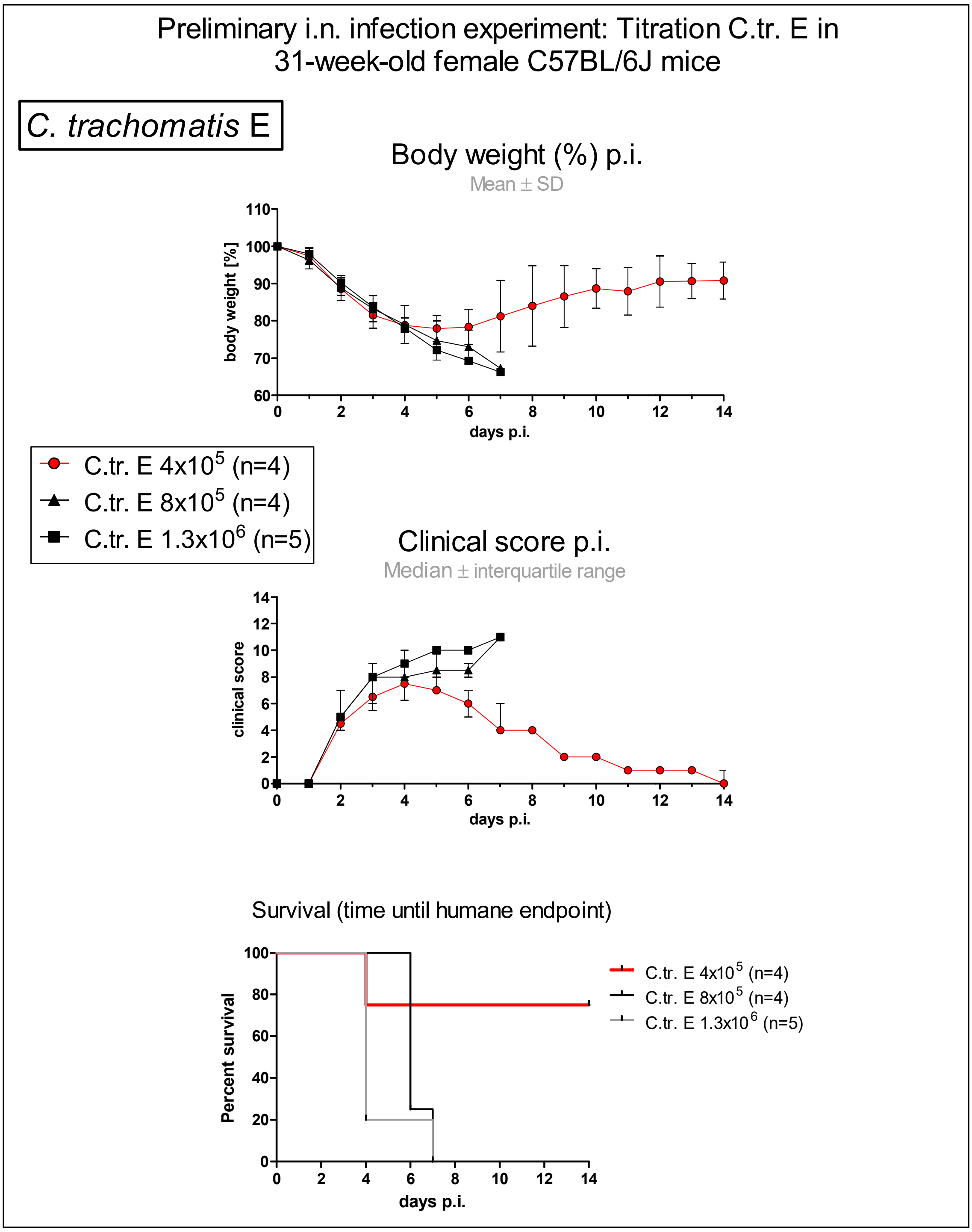

Figure S4. Body weight, clinical score, and Kaplan-Meier-Plot (survival) of an orientating i.n. C.tr. titration experiment for identification of the optimal amount of serovar E using 31-week-old female, hormone-synchronized mice. In preparation for the mouse lung infection used in the long-term protection model (see Material & Methods and Figure 1c), 31-week-old female hormone-synchronized C57BL/6J mice were infected i.n. with different IFU amounts of C.tr. serovar E (as indicated next to the graph and in the box). During the following 14 days, mice were monitored closely and body weight [%] (upper panel, mean ± standard deviation) and clinical score (middle panel, median ± interquartile range) were assessed daily. Mice were euthanized painlessly should humane endpoint criteria be reached (see Suppl. Material Table S1) at any point during the experiment. Survival rates [%] of the different IFU groups are depicted in a Kaplan-Meier-Plot (lower panel). Red data points and lines depict the C.tr. IFU later used in vaccination experiments.
